# Streptococcal Toxic Shock Syndrome: Persistent Diagnostic and Therapeutic Challenges

**DOI:** 10.7759/cureus.115467

**Published:** 2026-08-30

**Authors:** Jana Šimonová, Róbert Šimon, Ingrid Pirníková, Marek Andrejkovič, Peter Popaďák

**Affiliations:** 1 1st Department of Anesthesiology and Intensive Medicine, Faculty of Medicine, Pavol Jozef Šafárik University, Košice, SVK; 2 1st Department of Anesthesiology and Intensive Medicine, Louis Pasteur University Hospital, Košice, SVK; 3 1st Department of Surgery, Faculty of Medicine, Pavol Jozef Šafárik University, Košice, SVK; 4 1st Department of Surgery, Louis Pasteur University Hospital, Košice, SVK; 5 Department of Medical and Clinical Microbiology, Faculty of Medicine, Pavol Jozef Šafárik University, Košice, SVK; 6 Department of Forensic Medicine, Health Care Surveillance Authority, Košice, SVK

**Keywords:** bacterial translocation, emm1 genotype, septic shock, streptococcal toxic shock syndrome, streptococcus pyogenes

## Abstract

Toxic shock syndrome caused by *Streptococcus pyogenes* is a rare but life-threatening condition characterized by rapid clinical deterioration and high mortality. The *emm1* genotype is particularly virulent and drives a massive cytokine storm leading to multiorgan failure. Early diagnosis and aggressive multidisciplinary management are essential for patient survival.

A 36-year-old woman with a previously undiagnosed lymphoproliferative disorder and chronic leukopenia presented with a three-day history of fever, chest pain, diarrhea, and rapidly progressive cutaneous changes, including phlegmon and bullae. On admission, she was in septic shock with severe multiorgan dysfunction. Resuscitation was initiated immediately with large-volume crystalloids, vasopressors, and broad-spectrum antibiotics, including clindamycin to suppress toxin production. Despite escalating life support, she died within 12 hours of admission. Blood cultures yielded *S. pyogenes* *emm1*. Autopsy revealed unexpected polymicrobial meningitis involving *Neisseria meningitidis*, *S. pyogenes*, and hemolytic *Escherichia coli*. This postmortem finding was attributed to terminal bacterial translocation across a disrupted blood-brain barrier caused by the massive cytokine storm, rather than to an independent concurrent infection.

This case illustrates the extreme speed of clinical deterioration in streptococcal toxic shock syndrome driven by the *emm1* genotype. It highlights the necessity of initiating treatment based on clinical suspicion and the diagnostic complexities introduced by secondary postmortem microbiological findings in the setting of profound shock. It also underscores the importance of considering underlying immune deficiencies in patients presenting with fulminant streptococcal disease.

## Introduction

Toxic shock syndrome (TSS) is a rare but life-threatening condition caused by toxin-producing bacteria, most commonly *Staphylococcus aureus* and *Streptococcus pyogenes*. These organisms produce superantigenic exotoxins that trigger massive cytokine release, causing circulatory collapse and multiorgan dysfunction.

Historically, TSS was predominantly associated with *S. aureus* and the use of high-absorbency tampons in menstruating women. Following the removal of these specific products from the market, the epidemiological profile has shifted, making the recognition of nonmenstrual cases increasingly critical. In the United States, the annual incidence of TSS is estimated to be approximately 0.8 to 3.4 cases per 100,000 population [1]. TSS caused by streptococci carries a significantly higher mortality; the case fatality rate of streptococcal toxic shock syndrome (STSS) may exceed 50%, particularly with delayed diagnosis [1,2].

STSS typically arises from soft-tissue or cutaneous infections but may develop from any primary site. Early manifestations are nonspecific and may resemble an influenza-like illness, followed by rapid progression to shock and multiorgan failure [3,4]. Early diagnosis is essential and requires the integration of clinical findings with microbiological evidence. Management demands immediate intravenous fluid resuscitation, early broad-spectrum antimicrobial therapy, and urgent surgical source control when indicated. Clindamycin is routinely added to suppress toxin production, and intravenous immunoglobulin (IVIG) may be considered in severe cases. Despite aggressive treatment, death may occur within hours of presentation [1,3,4].

Given the rapid clinical deterioration and high mortality associated with this condition, reporting specific cases is crucial for improving clinical recognition. In this report, we present a unique case of STSS in a young woman who died within 12 hours of admission despite aggressive treatment.

## Case presentation

A 36-year-old woman with a history of breast reduction surgery and chronic leukopenia since 2017 without prior clinical consequences presented with a three-day history of fever, chills, diarrhea, vomiting, and burning pain on the left side of the chest radiating toward the abdomen. She reported dyspnea but denied headache and photophobia. Notably, she had been caring for her spouse, who had an upper respiratory tract infection in the days preceding her symptom onset, suggesting a possible source of bacterial or viral transmission. At the time of the patient's admission, her husband had already fully recovered.

On arrival, she was conscious and oriented, with no signs of meningeal irritation or focal neurological deficits. Her vital signs were as follows: heart rate, 124 beats per minute; blood pressure, 65/44 mmHg; and oxygen saturation, 90% on room air. The lungs were clear to auscultation. Abdominal tenderness was diffuse. Marked cutaneous changes were present over the left thoracic and abdominal wall, including phlegmon and bullae (Figure 1).

**Figure 1:**
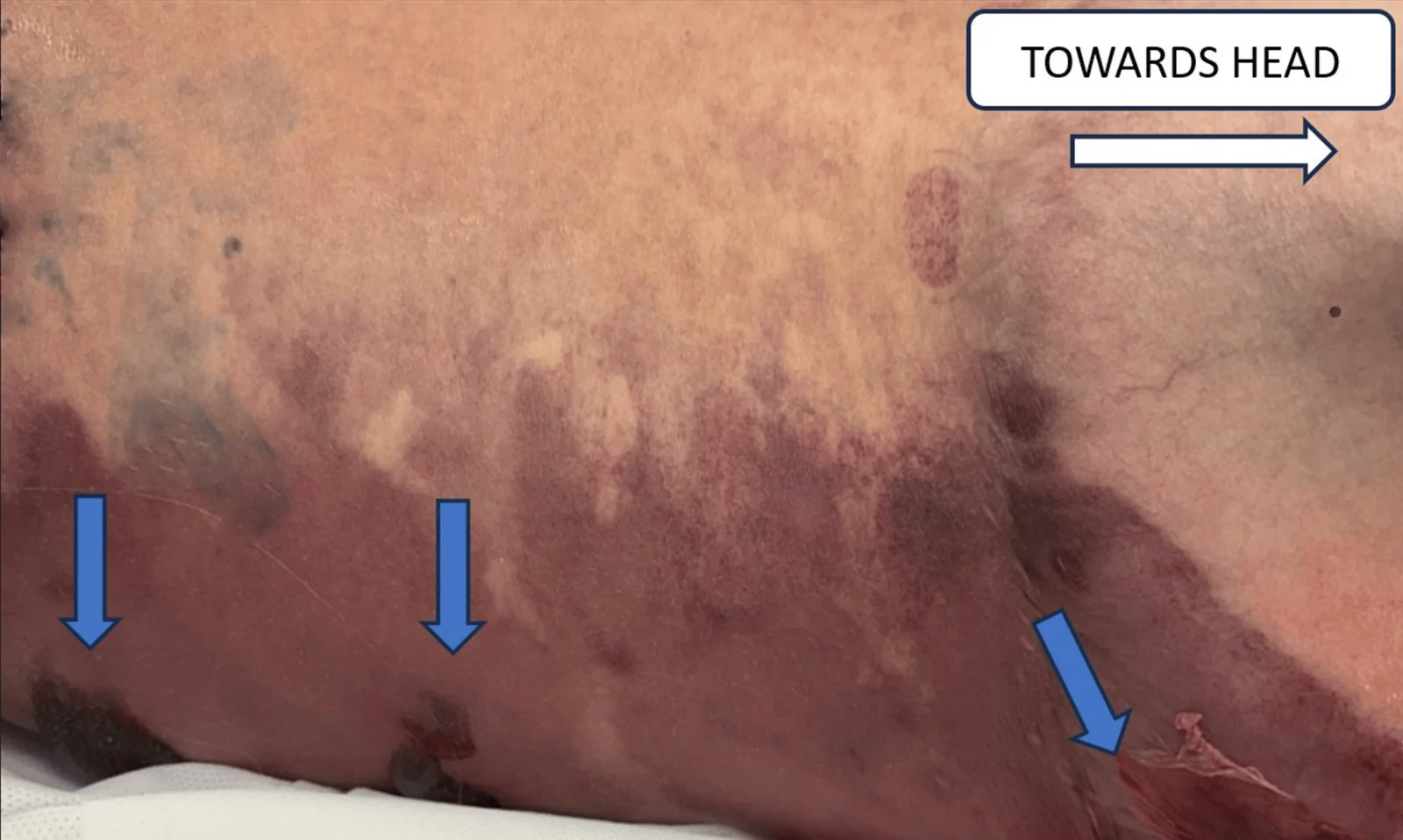
Phlegmonous Soft-Tissue Changes on Admission Phlegmonous soft-tissue changes with bullae (blue arrows) on the patient's back.

Laboratory studies on admission demonstrated markedly elevated serum C-reactive protein (CRP) and procalcitonin (PCT), leukopenia, elevated serum creatinine and creatine kinase, and a mildly prolonged international normalized ratio (INR), consistent with early multiorgan dysfunction (Table 1). A SARS-CoV-2 PCR test was negative.

**Table 1:** Laboratory Findings on Admission CRP: C-reactive protein; INR: international normalized ratio; PCT: procalcitonin

Parameter	Value	Reference range
CRP	453.04 mg/L	< 5 mg/L
Procalcitonin	19.38 ng/mL	< 0.5 ng/mL
Urea	10.84 mmol/L	2.5-7.8 mmol/L
Creatinine	240 µmol/L	44-97 µmol/L
Creatine kinase	10.34 µkat/L	< 2.55 µkat/L
Leucocyte count	2.39 × 10⁹/L	4.0-11.0 × 10⁹/L
Neutrophils	74.4%	33-80%
Eosinophils	1.7%	0-5%
Basophils	0	0-2%
Monocytes	0.8%	3-11%
Lymphocytes	9.9%	15-40%
Neutrophil-to-lymphocyte ratio	7.5	0-2.99
INR	1.44	0.85-1.15

Chest radiography performed on admission revealed no pulmonary infiltrates. Abdominal ultrasonography suggested changes consistent with sigmoid and descending colitis. Based on the clinical presentation, the patient was admitted to the surgical intensive care unit with a presumptive diagnosis of septic shock. At that time, the patient’s Sequential Organ Failure Assessment (SOFA) score was 10, indicating severe multiorgan dysfunction and an estimated mortality risk of 50% (Table 2) [5].

**Table 2:** Sequential Organ Failure Assessment (SOFA) Score on Admission The patient’s SOFA score on admission indicates multiple organ failure. paO_2_/FiO_2_: Horowitz index; GCS: Glasgow Coma Scale; MAP: mean arterial pressure; NE: norepinephrine

Parameter	Value	Normal value (0 point)	Points
paO_2_/FiO_2_	208.5 (no mechanical ventilation)	400	+2
Platelets count	113 x 10^3^/uL	>150 x 10^3^/uL	+1
GCS	13-14	15	+1
Bilirubin total	9.28 umol/L	<20 umol/L	0
MAP or vasoactive agent required	NE >0.1 mcg/kg/min	≥70 mmHg	+4
Creatinine	240 umol/L	<110 umol/L	+2

STSS was included among the primary differential diagnoses from the outset, given the combination of septic shock, soft-tissue involvement, and rapidly progressive clinical deterioration. Other differential diagnoses included necrotizing fasciitis, Gram-negative sepsis of colonic origin, and staphylococcal toxic shock syndrome.

Treatment and clinical course

Resuscitation was initiated immediately with a large-volume crystalloid infusion administered according to Surviving Sepsis Campaign guidelines. Norepinephrine was started via a central venous catheter to manage refractory hypotension. Blood cultures were obtained prior to antimicrobial administration, after which broad-spectrum intravenous therapy was initiated with meropenem (2 g every eight hours) and linezolid (600 mg every 12 hours). Given the clinical suspicion of STSS, clindamycin (900 mg every eight hours) was added to suppress streptococcal toxin production.

Despite an escalating norepinephrine dose (>0.2 µg/kg/min), the patient's hemodynamic status continued to deteriorate, prompting the addition of vasopressin to the vasopressor regimen. Consequently, the patient was transferred from the surgical intensive care unit to the Department of Anesthesiology and Intensive Medicine within one hour for advanced organ support. A subsequent computed tomography (CT) scan of the chest and abdomen, performed two hours after admission, demonstrated a large left pulmonary infiltrate, bilateral pleural effusions, left axillary lymphadenopathy, left thoracic and abdominal wall phlegmon, and early ischemic changes involving the liver, spleen, and kidneys (Figures 2-3). An intra-abdominal infection was ruled out; thus, surgical source control was not indicated, and operative intervention was deferred.

**Figure 2:**
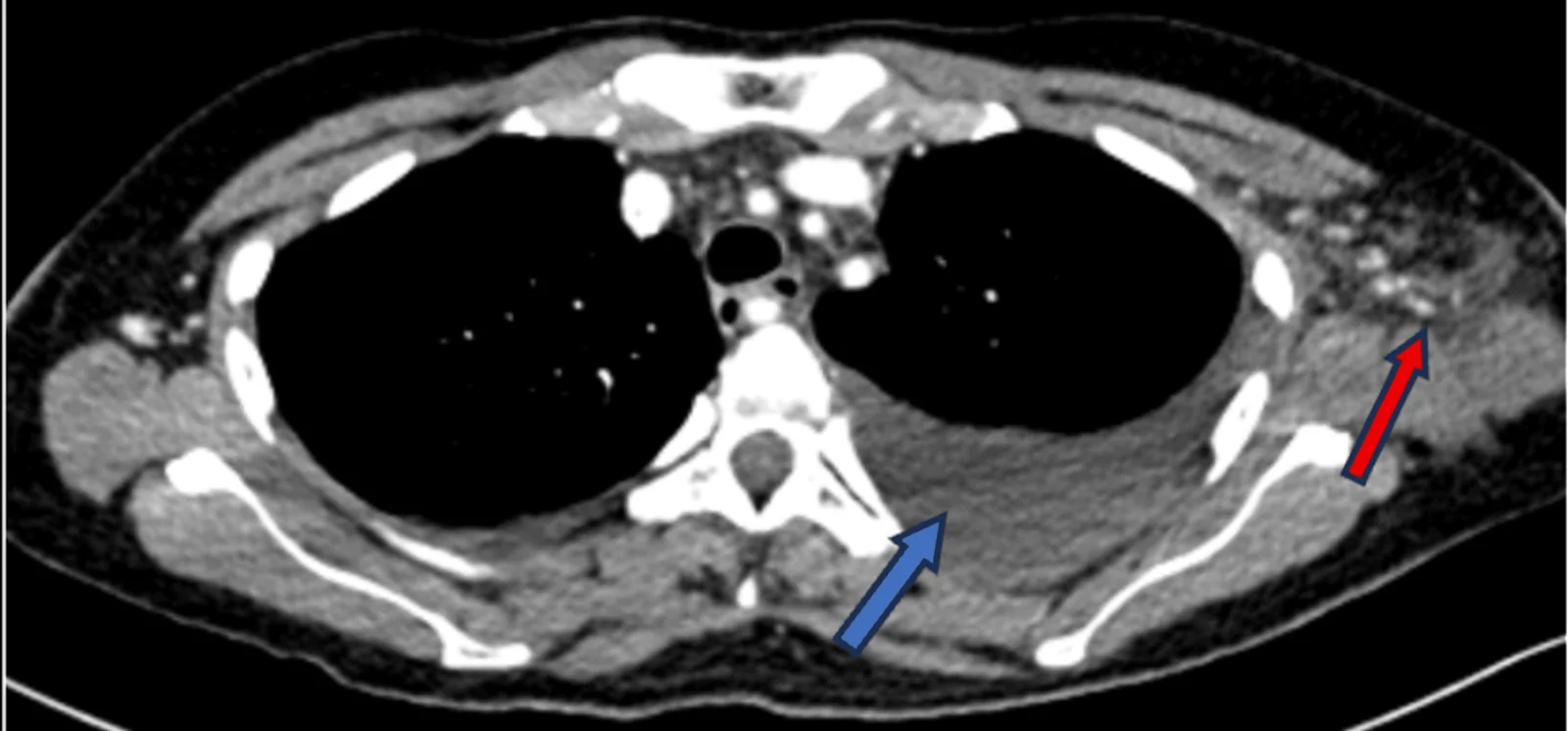
Axial CT Scan of the Chest Chest CT scan demonstrating a left-sided pleural effusion (blue arrow) and inflammatory changes in the left chest wall (red arrow). CT: computed tomography

**Figure 3:**
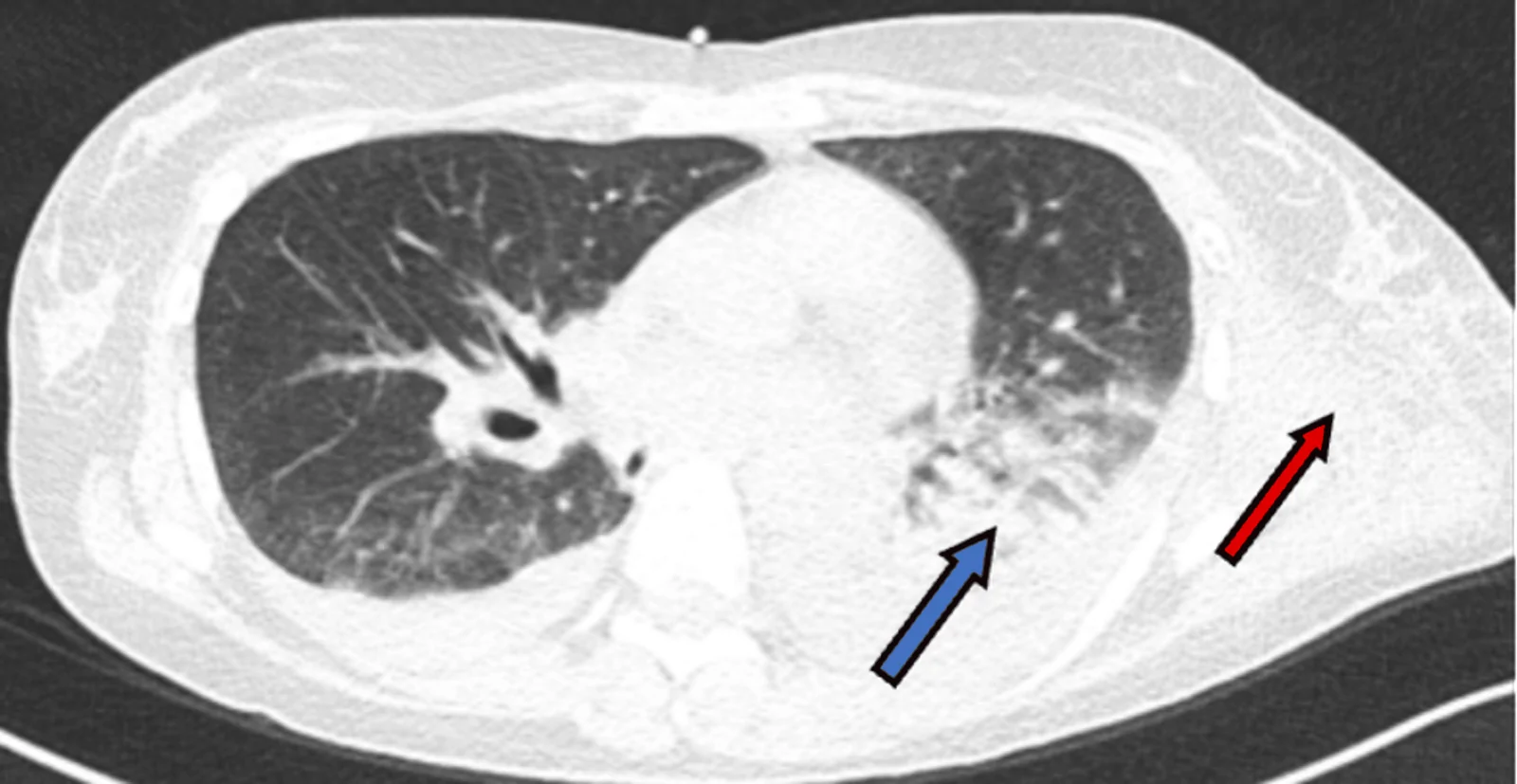
Axial CT Scan of the Chest Axial chest CT scan demonstrating infiltrative changes in the left lung parenchyma (blue arrow) and adjacent soft tissue (red arrow). CT: computed tomography

Urine output declined progressively, indicating evolving acute kidney injury. Hypoglycemia developed, requiring continuous intravenous glucose supplementation. Lower extremity sensory impairment prompted an urgent neurological assessment; magnetic resonance imaging (MRI) of the spine excluded myelopathy. Cerebrospinal fluid analysis was planned for the following morning. Worsening respiratory failure necessitated endotracheal intubation and mechanical ventilation. The soft-tissue lesions progressed rapidly during hospitalization (Figure 4).

**Figure 4:**
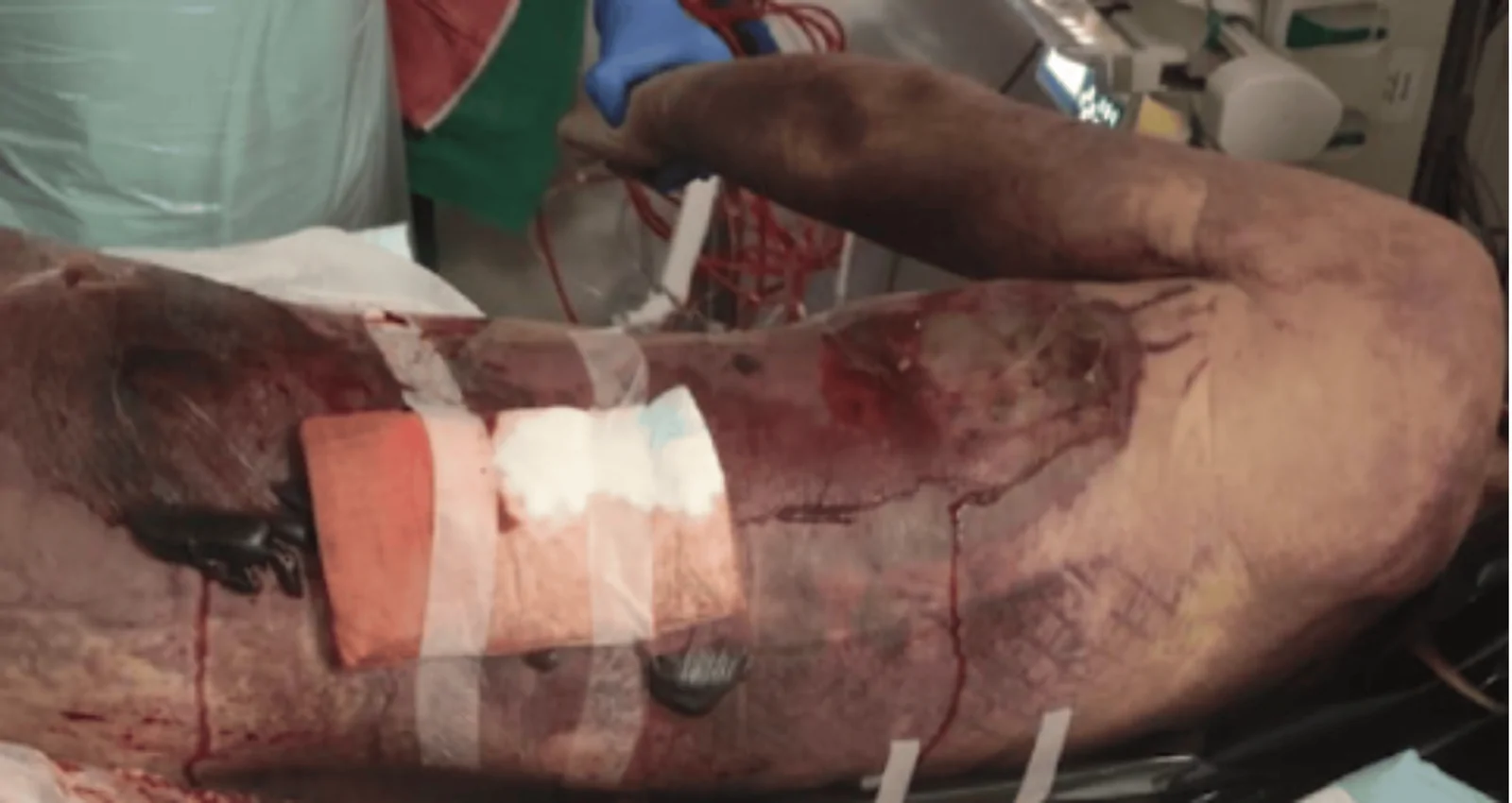
Progression of Soft-Tissue Manifestations (Approximately 10 Hours Post-admission)

Despite maximal supportive therapy, the patient deteriorated rapidly and died approximately 12 hours after admission from refractory septic shock and fulminant multiorgan failure. Consequently, the brief clinical course precluded the administration of adjunctive therapies beyond immediate resuscitation, such as IVIG.

Microbiological findings

Multiple clinical specimens were collected for microbiological analysis before and during antimicrobial therapy. Two blood culture sets from the central venous catheter (aerobic, anaerobic, and fungal vials; BD BACTEC; Becton, Dickinson and Company, Franklin Lakes, NJ, USA) were obtained. Samples were collected at the First Department of Surgery and subsequently at the First Department of Anesthesiology and Intensive Medicine. Additionally, oral and rectal swabs, as well as a urine sample, were collected at the First Department of Anesthesiology and Intensive Medicine. All samples were processed at the Department of Medical and Clinical Microbiology in accordance with standard laboratory operating procedures.

Blood cultures drawn from the central venous catheter immediately after placement were processed in an automated BD BACTEC FX200 system following the standard incubation protocol. The system signaled positivity 2 hours and 10 minutes after the vials were loaded. Positive blood cultures were subcultured onto Columbia agar with 5% sheep blood, MacConkey agar, Sabouraud agar, and chocolate agar. Smears from positive blood cultures were stained using a Gram stain. Microscopic examination revealed Gram-positive cocci arranged in chains. This finding was immediately reported to the attending physician. After overnight incubation, beta-hemolytic colonies were identified by mass spectrometry (MALDI Biotyper, Bruker Daltonics GmbH & Co. KG, Bremen, Germany) as *S. pyogenes*. Antimicrobial susceptibility testing performed according to current European Committee on Antimicrobial Susceptibility Testing (EUCAST) guidelines demonstrated susceptibility to all agents tested, including penicillin, ampicillin, amoxicillin-clavulanate, erythromycin, and clindamycin (breakpoint tables for interpretation of MICs and zone diameters, version 15.0, 2025). Molecular characterization at the National Reference Laboratory for Streptococcal Infections confirmed the presence of the *emm1* gene, identifying this as a high-virulence strain. The *emm1* genotype is associated with enhanced superantigen production and increased disease severity and has been identified as the dominant invasive strain in multiple European countries following the post-COVID-19 resurgence of invasive *S. pyogenes* infections [6].

An oral swab yielded both *S. pyogenes* carrying the *emm1* gene and non-PVL-producing *S. aureus *(negative for Panton-Valentine leukocidin). This finding suggests the oropharynx as a probable reservoir and potential primary portal of entry. A urine specimen grew hemolytic *Escherichia coli *(identified using the MALDI Biotyper, Bruker Daltonics GmbH & Co. KG), and by examination of beta-hemolysis on Columbia agar with 5% sheep blood) that was susceptible to all tested antimicrobial agents. Given the clinical context, the hemolytic *E. coli* isolate was not considered the primary pathogen.

The isolation of *S. pyogenes emm1* from a normally sterile site (blood), in combination with the clinical criteria of persistent hypotension, bilateral pleural effusions with pulmonary infiltrates, acute kidney injury, and soft-tissue infection, fulfilled the Centers for Disease Control and Prevention (CDC) criteria for a confirmed diagnosis of STSS [2].

Postmortem findings

Postmortem microbiological evaluation - comprising light microscopy at 1,000× magnification, bacterial culture, and rapid agglutination assays-revealed a polymicrobial infection. Specifically, *Neisseria meningitidis* serogroup C and heavy growth of *S. pyogenes* were abundantly isolated from the cerebrospinal fluid and brain tissue, while hemolytic *E. coli *was additionally detected in the CSF and on the meningeal surfaces. Extracranial findings included early *S. pyogenes*-associated inflammatory changes in the left lung, a left chest wall phlegmon attributable to *S. pyogenes*, and discrete colitis.

Importantly, the autopsy also revealed a previously undiagnosed lymphoproliferative disorder characterized by renal infiltration, generalized lymphadenopathy, and hypercellular bone marrow. Formal hematopathologic classification was not completed at autopsy; the omission of immunophenotyping represents a limitation of the postmortem workup. Nevertheless, this underlying condition, in conjunction with her history of chronic leukopenia, likely contributed to an immunocompromised state, increasing the patient's susceptibility to invasive bacterial infection.

## Discussion

STSS is an acute and severe life-threatening complication of predominantly invasive group A streptococcal (GAS) infections [7]. The central pathogen in this case is S*. pyogenes emm1*, which drove the fulminant clinical course from the outset. The remaining microorganisms identified across clinical specimens - *S. aureus* in the oral cavity, hemolytic *E. coli* in the urinary tract, and *N. meningitidis* found postmortem in the cerebrospinal fluid and brain - reflect either incidental colonization or secondary phenomena arising in the context of overwhelming streptococcal sepsis and profound immune failure. Distinguishing the causative pathogen from bystander flora is critical to maintaining diagnostic clarity in cases of this complexity.

STSS is a severe, rapidly progressive condition with a fatal outcome if left untreated. *S. pyogenes* produces superantigenic exotoxins that activate T lymphocytes by binding directly to T-cell receptors while simultaneously engaging antigen-presenting cells through MHC class II molecules [8,9]. This leads to massive cytokine release - a so-called cytokine storm [10,11] - involving interferon-gamma, TNF-α, and interleukins [1,2,6,8], resulting in shock, multiorgan dysfunction syndrome (MODS), and death within hours of symptom onset. The *emm1* genotype is recognized as a particularly virulent strain associated with greater superantigen production and higher case fatality rates in epidemiological studies.

While STSS can occur in young, otherwise healthy individuals, established risk factors include diabetes mellitus, advanced age, breaches in skin integrity, and immunocompromised states. In our patient, the previously undiagnosed lymphoproliferative disorder identified at autopsy, along with documented chronic leukopenia, provides critical context. Together, these underlying conditions likely induced a state of latent immunosuppression, explaining the patient's heightened vulnerability to invasive streptococcal infection, the inability to mount an effective immune response, and the rapid progression to overwhelming exotoxin-mediated shock.

The diagnostic criteria established by the CDC require isolation of *S. pyogenes *from a normally sterile site to confirm STSS, combined with hypotension and at least two of the following: renal impairment, coagulopathy, hepatic dysfunction, respiratory failure, or soft-tissue necrosis. This patient fulfilled both laboratory and clinical criteria: *S. pyogenes emm1 *was isolated from blood cultures and subsequently from cerebrospinal fluid and brain tissue postmortem; persistent refractory hypotension was documented throughout; and CT confirmed bilateral pleural effusions, pulmonary infiltrates, and early organ ischemia consistent with MODS. Objective assessment using the SOFA score confirmed severe multiorgan dysfunction on admission, consistent with the observed clinical trajectory.

Identifying whether the primary infection source was respiratory or soft tissue remains a diagnostic challenge. Given the patient's history (close contact with her symptomatic spouse), microbiological findings (*S. pyogenes* detected in the oral swab), and clinical course, an upper respiratory tract infection was likely the primary site, which - exacerbated by documented immunocompromise - rapidly progressed to the lungs and disseminated hematogenously to soft tissues and distant organs. Subsequent massive bacterial toxin production precipitated refractory septic shock. Notably, there was no documented history of antecedent trauma or skin injury.

The first limitation of the clinical workup was the omission of a skin lesion culture. However, as previously noted, the cutaneous lesions observed on admission were initially interpreted as secondary manifestations of septic shock resulting from profound vasoconstriction, rather than a primary infectious focus. Nevertheless, postmortem evaluation ultimately demonstrated the presence of *S. pyogenes* within the soft tissues.

The next diagnostically challenging aspect of this case is the postmortem detection of *N. meningitidis* serogroup C. Postmortem microbiological evaluation - comprising light microscopy at 1,000× magnification, bacterial culture, and rapid agglutination assays - demonstrated this organism alongside heavy growth of *S. pyogenes* in the cerebrospinal fluid, brain tissue, and on the meningeal surfaces; PCR testing was not performed. However, several lines of evidence argue strongly against *N. meningitidis* as a causally relevant pathogen in this patient. Throughout her entire clinical course, the patient remained conscious and oriented, with no headache, photophobia, neck stiffness, or other signs of meningeal irritation - findings that are virtually invariably present in clinically manifest meningococcal meningitis. Furthermore, all blood culture sets collected prior to antimicrobial therapy signaled positivity within approximately two hours of vial loading and yielded exclusively *S. pyogenes*; *N. meningitidis* was not isolated from any antemortem specimen. This early and exclusive bacteremia with *S. pyogenes* across all submitted culture sets is highly consistent with the clinical presentation and directly supports STSS as the primary diagnosis. Crucially, the *emm1* genotype identified in this case encodes a toxigenic M protein with well-characterized superantigenic and antiphagocytic properties, providing a clear pathobiological mechanism that accounts fully for the observed clinical trajectory of rapid septic shock and multiorgan failure without invoking an additional causal pathogen.

The postmortem finding of *N. meningitidis* is most plausibly explained by translocation from the nasopharynx - where asymptomatic carriage is estimated in 5% to 10% of the general population [12] - across a profoundly disrupted blood-brain barrier in the terminal phase of overwhelming STSS. The massive cytokine storm characteristic of STSS damages endothelial tight junctions and increases blood-brain barrier permeability, facilitating the paracellular passage of colonizing organisms without eliciting a clinically recognizable meningitic syndrome [13,14]. Postmortem bacterial translocation or autopsy-related contamination represents an additional alternative that cannot be excluded. It must be acknowledged that no nasopharyngeal swab was obtained during hospitalization; this represents a second limitation of the clinical workup, as such a specimen could have confirmed or refuted asymptomatic carriage and thereby strengthened the interpretive argument. Nevertheless, the totality of clinical and microbiological evidence identifies *S. pyogenes* *emm1* as the sole causal agent, and the postmortem polymicrobial meningeal findings should be interpreted as a terminal or postmortem phenomenon rather than an independent clinical infection.

Based on established literature, clindamycin was added to the initial empiric antimicrobial regimen to suppress toxin production in the setting of suspected STSS, prior to microbiological confirmation [1,3,4]. However, increasing clindamycin resistance in GAS has called into question its utility for this indication. Linezolid also inhibits GAS toxin and virulence factor production, but clinical efficacy data remain sparse [15]. Surgical evaluation was performed, and an intra-abdominal infectious source was excluded by CT. The absence of a surgically accessible focus meant that operative source control was not feasible. IVIG, which may modulate the superantigen-driven immune response in STSS, was not administered. The extreme rapidity of clinical deterioration - with death occurring within 12 hours of admission - precluded its initiation, as there was no opportunity to introduce adjunctive therapies beyond immediate resuscitation. Additionally, STSS was not confirmed microbiologically at the time of the clinical decision, and IVIG would not be appropriate across all differential diagnoses considered on admission. A small, randomized, placebo-controlled trial of IVIG in STSS demonstrated a reduction in mortality among patients receiving IVIG compared with those receiving antibiotics alone [16].

Consistent with other recently published reports highlighting nonspecific early symptoms, such as chest wall or abdominal pain [17,18], our case demonstrates the extreme diagnostic complexity and rapid clinical deterioration inherent to STSS, often necessitating empiric toxin-suppressing therapy prior to microbiological confirmation.

## Conclusions

STSS caused by the *emm1 *genotype of *S. pyogenes *remains a rare but rapidly fatal condition that demands immediate clinical recognition and aggressive multidisciplinary management.

This case illustrates the extreme speed of clinical deterioration, the critical importance of initiating empiric treatment based on clinical suspicion without awaiting microbiological confirmation, and the diagnostic complexity introduced by secondary postmortem microbiological findings in overwhelming septic shock. Furthermore, the postmortem discovery of an undiagnosed lymphoproliferative disorder underscores the need to consider underlying immune deficiencies in patients presenting with unusually severe or rapidly progressive streptococcal disease.

## References

[1] Ross A, Shoff HW (2025). Toxic shock syndrome. StatPearls [Internet].

[2] (2010). Streptococcal toxic shock syndrome (STSS) 2010 case definition. https://ndc.services.cdc.gov/case-definitions/streptococcal-toxic-shock-syndrome-2010/.

[3] Atchade E, De Tymowski C, Grall N, Tanaka S, Montravers P (2024). Toxic shock syndrome: a literature review. Antibiotics (Basel).

[4] Stevens DL (2002). Streptococcal toxic shock syndrome. Clin Microbiol Infect.

[5] Vincent JL, Moreno R, Takala J (1996). The SOFA (Sepsis-related Organ Failure Assessment) score to describe organ dysfunction/failure. On behalf of the Working Group on Sepsis-Related Problems of the European Society of Intensive Care Medicine. Intensive Care Med.

[6] Goldberg-Bockhorn E, Hagemann B, Furitsch M, Hoffmann TK (2024). Invasive group A streptococcal infections in Europe after the COVID-19 pandemic. Dtsch Arztebl Int.

[7] Bartoszko JJ, Elias Z, Rudziak P, Lo CK, Thabane L, Mertz D, Loeb M (2022). Prognostic factors for streptococcal toxic shock syndrome: systematic review and meta-analysis. BMJ Open.

[8] Li H, Llera A, Malchiodi EL, Mariuzza RA (1999). The structural basis of T cell activation by superantigens. Annu Rev Immunol.

[9] Sundberg EJ, Andersen PS, Schlievert PM (2003). Structural, energetic, and functional analysis of a protein-protein interface at distinct stages of affinity maturation. Structure.

[10] Bueno C, Criado G, McCormick JK, Madrenas J (2007). T cell signalling induced by bacterial superantigens. Chem Immunol Allergy.

[11] Dinarello CA (2000). Proinflammatory cytokines. Chest.

[12] Christensen H, May M, Bowen L (2010). Meningococcal carriage by age: a systematic review and meta-analysis. Lancet Infect Dis.

[13] Sellner J, Leib SL (2006). In bacterial meningitis cortical brain damage is associated with changes in parenchymal MMP-9/TIMP-1 ratio and increased collagen type IV degradation. Neurobiol Dis.

[14] Moreillon P, Majcherczyk PA (2003). Proinflammatory activity of cell-wall constituents from gram-positive bacteria. Scand J Infect Dis.

[15] Babiker A, Warner S, Li X (2025). Adjunctive linezolid versus clindamycin for toxin inhibition in β-lactam-treated patients with invasive group A streptococcal infections in 195 US hospitals from 2016 to 2021: a retrospective cohort study with target trial emulation. Lancet Infect Dis.

[16] Lappin E, Ferguson AJ (2009). Gram-positive toxic shock syndromes. Lancet Infect Dis.

[17] Boyko ME (2025). Streptococcal toxic shock syndrome: a case report highlighting the importance of early recognition and intervention. Cureus.

[18] Kurokawa R, Kurokawa M, Onoda S, Onozato Y, Nakajima A, Wada T, Abe O (2025). Early diagnosis of streptococcal toxic shock syndrome with abdominal computed tomography: a case report. Cureus.

